# Translation, Adaptation, and Validation of a Multitasking Instrument in the Context of Collectivist Asian Culture

**DOI:** 10.11621/pir.2022.0109

**Published:** 2022-03-30

**Authors:** Saima Kalsoom, Anila Kamal

**Affiliations:** a Department of Professional Psychology, Bahira University, Islamabad, Pakistan; b Rawalpindi Women University, Rawalpindi, Pakistan & National Institute of Psychology, Quaid-i-Azam University, Islamabad, Pakistan

**Keywords:** Multitasking measure, empirical validity, construct validity, perceived multitasking ability, adaptation and validation

## Abstract

**Background:**

Multitasking is a rapidly evolving construct and we are in dire need of a sound tool for measuring multitasking behaviors and abilities across socio-cultural contexts. To this end, this study has put forward a cultural adaptation (through back translation) of an already developed (Kushniryk, 2008) measure i.e., Communication Specific Multitasking Measurement Instrument.

**Objective:**

This study is intended to translate, adapt, and validate a multitasking measure i.e., Communication Specific Multitasking Measurement Instrument (CSMMI; Kushniryk, 2008) in the context of collectivist culture in Pakistan.

**Design:**

The study was composed of two parts. The first part was completed in two phases. Phase I employed back and forward translation methods to translate the multitasking measure into an indigenous language. Phase II provided empirical validity of the translated and adapted instrument (CSMMI) using exploratory factor analysis (EFA) on data collected from a sample of 230 married individuals. The second part of the study was designed to establish construct validity of the translated instrument using confirmatory factor analysis (CFA) on a larger data set of married individuals.

**Results:**

EFA using a varimax rotation on all 19 items of CSMMI showed that the instrument is a three-dimensional measure. CFA confirmed that the translated and adapted instrument is also a three-dimensional measure on the larger data set. Analysis of the intraclass correlation and alpha coefficient provided sound evidence for validity and reliability of the measure (CSMMI).

**Conclusion:**

The findings of this study indicate that the translated and adapted multitasking measure (CSMMI) is reliable and valid when applied to the culturally collectivist population of Pakistan. This also pertains to any other populations where the translation is adequately applicable.

## Introduction

The act of carrying out any two or more activities simultaneously is referred to as multitasking. The most common forms include watching television while eating, walking while eating, driving while talking on the phone, and typing while listening to music (Widyahastuti & Anwar, 2017). Some people believe that accomplishing several activities at once (multitasking) is a good thing and can increase productivity. In order to reach an adequate performance and achieve a certain goal, these people accept that multitasking is essentially required and may develop a preference for multitasking (Sanbonmatsu et al., 2013). Though multitasking is described as a behavior in which an individual is engaged in several tasks at the same time, it also refers to an ability to handle these tasks and to switch quickly between tasks if that is required for successful performance. Jarmon (2008) explained that multitasking can manifest in three ways. A person is either able to work on two or more tasks simultaneously, such as reading while watching television, switch between tasks repeatedly, such as alternating between answering e-mails and listening to discussions during class, or complete two or more tasks with speed and accuracy. Similarly, Kushniryk (2008) defined multitasking as completing a set of jobs within a certain time period, either simultaneously or with frequent and swift transitions between one task and another. Kushniryk (2008) also proposed multitasking as a multi-faceted construct. Namely, there are four facets to multitasking: general multitasking abilities, multitasking on a computer, the ability to perform two primary tasks simultaneously, and the ability to perform primary and secondary tasks simultaneously.

Organizations treat an ability to multitask as an essential element of any job description and a central demand in almost every job. Many researchers have stressed that an employee’s multitasking ability is entirely necessary for effective performance and productivity (Buhner et al., 2006; Sanderson et al., 2013). An inclination to multitasking is collective and thereby also individualistic (Hall, 1959: 1976). This inclination has been studied as both a cultural and individual phenomenon, touching on monochronicity and polychronicity within one culture (Lindquist & Kaufman, 2007; Poposki et al., 2009a). Multitasking is of normative value for many American organizations, where work schedule coordination has become custom in organizational culture, more so than within organizations in India (Hall, 1983; Lasane & O’Donnell, 1993; Palmer & Schoorman, 1999), which is a culturally similar to Pakistan. One Pakistani published study (Sehrish & Zubair, 2013) explored polychronicity, time management, and work-related quality of life among bank employees. Sehrish & Zubair explained that people are becoming more interested in performing multiple tasks simultaneously, tending towards multitasking. This was found to have a positive impact on their daily life, hence the need to explore multitasking in socio-organizational backgrounds across cultural contexts.

Multitasking encompasses aspects of communication, such as frequently talking on the phone while driving or surfing the web while listening to a lecture (Kushniryk, 2008). As multitasking is a complex and evolving construct, this study involves the translation and adaptation of an instrument, which is based on the degree of perceived multitasking ability concerning communication, for use in the cultural context of Pakistan. This instrument was developed (Kushniryk, 2008) based on the aforementioned definition: to accomplish multiple tasks/goals in the same general time period, either simultaneously or by engaging in frequent switches between individual tasks (Poposki & Oswald, 2010) alongside minimum communication based tasks. This instrument has also been translated and adapted into Chinees (Luo et al., 2018). Widyahastuti and Anwar (2017) used this instrument to study links between the Big Five personality dimensions and multitasking. The results of this study demonstrated that the dimensions, such as extraversion, conscientiousness, openness and neuroticism, do not significantly effect ones predisposition to multitasking. However, the results of another study (Kalsoom & Kamal, 2020) showed that CSMMI is correlated with Pakistani multitasking preferences, gender role attitudes, and marital adjustment.

Gender differences in the propensity towards multitasking is a very important contemporary aspect to investigate. Various studies (Bianchi, et al., 2006; Bianchi & Wight 2010; Galinsky at al., 2005; Offer & Schneider, 2011) have classed gender as an important variable while studying multitasking and have consistently found differences between men and women (Floro & Miles, 2003; Kushniryk, 2008; Mantyla, 2013) on multitasking measures. In their investigations, Mäntylä (2013), Offer & Schneider (2011), Ruiz (2013), and Stoet et al. (2013) reported women as having greater multitasking abilities than men in both work and home spheres. On the other hand, Buser & Peter (2012) suggested minimal gender differences with regards to multitasking ability and preferences. Offer and Schneider’s 2011 review presented a comprehensive and qualitative view of multitasking with respect to gender and gender roles in terms of both paid and unpaid work distribution. The results of another study revealed insignificant gender differences and rejected the common stereotype that women are better at multitasking (due to an ability to juggle various roles at work and in the home) than men, at least in the typical consecutive and concurrent/ simultaneous multitasking settings (Hirsch et al., 2019). In a recent empirical investigation, Lui et al. (2020) noted a smaller concurrent multitasking (dual-task) cost for men than women, and no gender difference when it came to sequential multitasking (task-switching) cost. Men had more experience engaging in multitasking that involved video games, whereas women were more experienced engaging in multitasking that involved instant messaging, music, and web surfing. The results can be interpreted as stemming from individual cognitive differences. Taking into account these studies, the current study also intends to establish contrasted group validity (through gender differences) of the measure, after completing the translation and adaptation process. This would be an important addition to the existing knowledge-base and a step towards understanding the construct of multitasking and the ways in which married men and women with children perceive and use their time, while occupying paid and unpaid roles simultaneously.

In a technology driven world, the nature of paid and unpaid work has become very diverse and complex. In this context, multitasking is considered an essential ingredient of our daily juggling of multiple roles. Therefore, multitasking is indispensable in every culture and setting, especially at work. This has created a need for an appropriate indigenous measure of multitasking that is applicable in collectivist Asian cultures such as that of Pakistan. The construct of multitasking was initially derived from computer science and has become an increasingly common human behavioral attribute. It is considered a skill and an essential ability, especially for working individuals. As Lindbeck & Snower (2000) and Milgrom & Roberts (1990) explained, individuals who like working in multitasking organizations are able to thrive in such environments and find positive meaning in their work. In addition to juggling multiple activities and tasks in the workplace, employees are engaged in family domains and both work and non-work related interactions occur (Greenhaus & Powell, 2006; Gutek et al., 1991). These work-family interactions may require even higher multitasking ability. In Pakistan, however, this aspect of time orientation has not yet been considered for empirical investigation. Therefore, this study focuses on first addressing this unmet need of measurement tool, as this is required to empirically investigate the multitasking abilities of the individuals from a collectivist cultural perspective as opposed to a western individualist perspective.

Pakistani society is collectivist in nature — a nature rooted in its cultural traditions, values and customs. However, globalization, technological advancement, the expansion of education, and exposure to the media have caused these aspects of Pakistani culture to rapidly evolve. One of the ways in which this has manifested is in a growing need for multitasking. Increased urbanization has led to an increase in the number of women in work and in the demand for multiplicity of roles. Moreover, economic instability and inflation have increased, as have working hours, and it has become a necessity for most people in Asia, particularly in Pakistan, to have more than one source of income. People are balancing two or more paid roles, alongside personal and familial responsibilities, and this has created a need to study multitasking behavior and attitudes among married men and women, with children. The evolving construct of multitasking is relatively new in the Asian literature, specifically in context of Pakistan. Only one published study (Sehrish & Zubair, 2013) is available that discusses polychronicity and time management by using the 4-item scale, the Polychronic Attitude Index (PAI) in English. Hence there exists a need to translate, adapt, and validate a recently developed and applicable measure, in order to provide a comprehensive multitasking measure for socially and culturally collectivist populations. To meet this need, two studies were devised to target the translation, adaptation, and validity (both empirical and construct) of the Communication Specific Multitasking Measurement Instrument (CSMMI) developed by Kushniryk (2008).

## Method

The current study was completed into two parts. Study I tackled translation, adaption, and empirical validation through exploratory factor analysis (EFA) of the multitasking instrument. Study II was concerned with construct validation though confirmatory factor analysis (CFA), contrasted group validity, and reliability of the translated and adapted measure.

## Study I

The main objectives of this study were twofold. First, to translate and adapt the Communication Specific Multitasking Measurement Instrument (CSMMI) into Urdu, the national language of Pakistan, from its original English. Second, to empirically validate (through EFA) the translated and adapted version of CSMMI. These two objectives were achieved into two phases, respectively.

### Phase I: Translation and Adaptation of Multitasking Instrument

In order to achieve the first objectives of Study I, the translation and adaptation of CSMMI was completed following the guidelines given by Brislin (1976; 1980), Hambleton (1994), and Sousa & Rojjanasrirat (2011). Forward and back translation methods were employed by five bilingual expert translators for the translation of of all the 19 items of CSMMI. Subject matter expert method (SME) was used for the sorting of translated items. Two slight modifications were made in two items of CSMMI. To the phrase in item 1, “I like talking on the phone while I am driving my car”, were added *bicycle* and *any vehicle*, so the phrase became: “I like talking on the phone while I am driving my car/bicycle/any vehicle”, making it more general. Similarly, the phrase *during class lecture* was removed from the phase in item 4, “I can easily understand and comprehend material presented while I am doing something unrelated.” These changes were made in view of the consideration that the data of this study was to be collected from married men and women not university students. Therefore, it was optimal make the questionnaire more general for the intended target population. No item was excluded from the original measure and, upon completion of the translation and adaptation, all 19 items of the translated scale were used to collect the data for empirical validation.

### Phase II: Empirical validation

#### Participants

To achieve the second objective of Study I, a sample (230) of married men (*n* = 126) and women, both working (*n* = 61) and housewives (*n* = 43), were selected to evaluate the Urdu CSMMI. The ages of these individuals ranged from 20-62 years (*M* = 35.53 & *SD* = 8.40). All the participants of this study were selected from the twin cities i.e., Rawalpindi and Islamabad. Working individuals were approached one-by-one at their respective institutes and organizations in these two cities. Written permission from the concerned authorities of these organizations was also taken. Purposive and convenience sampling techniques were employed to select the sample for cross sectional data collection. Informed consent was taken, and confidentiality and anonymity were ensured.

#### Procedure

CSMMI is a five-point Likert type scale with the following response options: 1: *Strongly disagree*; 2: *Disagree*; 3: *Neither agree nor disagree*; 4: *Agree*; 5: *Strongly agree*. The scale originally consisted of 19 items and three facets i.e., general multitasking abilities, ability to perform two/ more than two primary tasks simultaneously, and the ability to perform primary and secondary tasks simultaneously. Seven items are reverse coded. The score range is 19-95, whereby a high scores indicated high perceived multitasking abilities and low scores indicate low perceived multitasking abilities. The alpha reliability reported by the original author is .82 (Kushniryk, 2008). Another study (Widyahastuti & Anwar, 2017) reported a similar value (.81) for alpha reliability, indicating that the measure is valid and reliable to use. To collect the data on the translated CSMMI, a separate demographic sheet was prepared to acquire information regarding gender, age, and work status of the participants. Willing participants were provided with the translated instrument, demographic information sheet, and informed consent. The data was collected individually through one-to-one interaction. Verbal and written informed consent of all the participants was taken. Participants were further assured that the confidentiality and anonymity of their data would be upheld.

## Results

### Empirical Validity through Exploration of Factor Structure

Factorial validity of translated and adapted measures is as crucial as for newly developed measures. In a recent study, Hair et al. (2019) put forward factorial validity as essential for proof of validity when using EFA to validate an attitude measure. Similarly, Püsküllüoğlu et al. (2014) followed the same approach (EFA & CFA) for establishing the psychometric properties of the translated measure. EFA provides sufficient empirical and construct validation for any measure to verify the findings across populations and cultures. McMurtry & Torres (2002) and Titlestad et al. (2017) suggested to use EFA followed by CFA to validate the factor structure of the translated instruments. The need and justification to explore the factor structure of the Pakistani CSMMI is based upon the logical ground of individual differences across socio-cultural contexts. Moreover, the original version of CSMMI was developed and validated based on a sample of potential incumbents, whereas the current study has collected data from a sample of employed and married individuals, which is significant when considering the cross-language construct validation. In order to determine the suitability of the original factor structure of CSMMI on the Pakistani sample, CFA was performed to replicate the original factor structure on the data collected for this study. The results of the CFA testing revealed that the original factor structure is not suitable in this case, as the model fit indices suggest a poor model fit for the adjusted scale. Hence, it was decided to explore the factorial structures of the Pakistani CSMMI theoretically. To achieve the second objective of Study I, factor structure of the translated and adapted versions of multitasking measure (CSMMI) was run by first employing EFA on the small data set of Study I and then CFA on the larger data set collected for Study II.

### Exploratory Factor Analysis (EFA)

The Kaiser-Meyer-Olkin (KMO) test was used to determine the suitability of the data for EFA. A KMO value of .75 sample adequacy indicated that the data was appropriate for factor analysis, based on the recommendation by Field (2009) that values between .7 and .8 are sufficient to carry out EFA. Bartlett’s Test of Sphericity was also conducted on the data and it was found that *p* = .000 which is below the .05 criteria. Therefore, EFA was performed to explore the factor structure for the translated items of CSMMI on the data of married individuals.

The scree plot suggested that factors I, II, and II are the predominent factors, showing eigenvalues of greater than 3, 2 and 1, respectively. In view of this, the three factors with eigenvalues greater than 1 were retained for the EFA. Factor I displayed an eigenvalue of 3.25 and represented 14.49% of the total variance, the highest among the three factors. Factor II had an eigenvalue of 2.47, representing 12.97% of variance, and factor III displayed an eigenvalue of 1.53 and represented 11.77% of the variance. Overall, these three factors accounted for 39.24% of the variance in the model.

**Figure 1.**
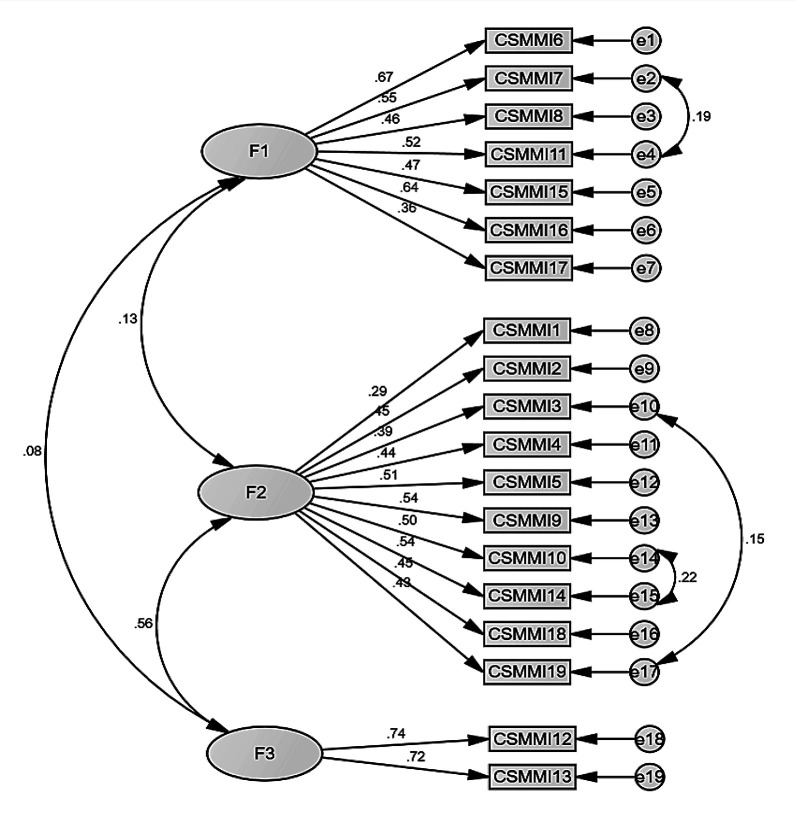
Measurement model and factor loading for the EFA/CFA on the 19 items of CSMMI (*N* = 850).

The results in *Table 1* display factor loadings of all the 19 items of CSMMI on the basis of their being greater than .40 (Field, 2013; Tabachnick & Fidell, 2013). *Figure 1* provides a visual representation of the measurement model. These factor loadings were obtained using the maximum likelihood method (ML) and Varimax rotation to determine the factor structure of the measures. Three factors were considered as three submeasures of CSMMI. The newly emerged factors are as follows:

1. General Multitasking Ability
2. The Ability to Perform two/more than two Primary Tasks/Activities Simultaneously
3. The ability to Perform Primary and Secondary Tasks Simultaneously

**Table 1:** Factor Loading of the Communication Specific Multitasking Measurement Instrument (CSMMI) through Principal Axis Factoring using the Maximum Likelihood Method (N = 230)

Serial No.	Item No.	GMA^a^	APTMTPTS^b^	APPSTS^c^	h2
1	16	**.53**	–.09	–.07	.68
2	6	**.70**	.05	.09	.73
3	7	**.44**	–.07	.03	.70
4	15	**.45**	–.01	–.04	.72
5	11	**.44**	.00	.25	.66
6	8	**.50**	.04	–.03	.76
7	17	**.51**	–.13	–.20	.56
8	3	–.16	**.47**	–.15	.54
9	5	–.05	**.44**	.08	.62
10	9	–.08	**.43**	.31	.61
11	2	–.08	**.58**	.18	.59
12	10	.23	**.48**	.25	.66
13	4	.15	**.41**	.16	.59
14	18	–.03	**.50**	–.05	.58
15	14	.17	**.45**	.20	.70
16	19	–.21	**.44**	.19	.61
17	1	.04	.**40**	.28	.58
18	12	.01	.05	**.74**	.70
19	13	–.12	.04	**.87**	.71

*Note. Factor Loading > .40 is reported for each factor; ^a^GMA = General Multitasking Ability; ^b^APTMTPTS= Ability to Perform Two/More Than Two Primary Tasks Simultaneously; ^c^APPSTS = Ability to Perform Primary and Secondary Tasks Simultaneously*.

The original measure contained four factors, whereas EFA on this study led to the emergence of three factors. The fourth factor was previously referred to (Kushniryk, 2008) as “computer multitasking ability”. Originally, this factor was comprised of two items. However, in the factor structure revealed in this study, these two items were loaded under the second factor i.e., the ability to perform two/more than two primary tasks simultaneously. Therefore, these two items were accounted for by the second factor and there was no need for a fourth factor to account for them.

The third factor in this factor sucture is estimated to have only two items out of a total 19. Conventionally, measures comprise multiple items and the maximum number of items per scale depends on the complexity of the variable being measured (Robinson, 2018). A researcher may be able to construct their own version, for example by selecting only a few items, but those with the highest factor loadings for that scale. In the same vein, the factor loadings for the two third factor items of CSMMI particularly signficiant within the 19-item measure. They contribute 7.50% variance to the overall model, and this should be noted. Moreover, these two items are comparable to factors suggested in the original measure. As this should not be ignored, the factor was retained as a submeasure. Neverthless, the original factor structure was determined (Kushniryk, 2008) from the data of undergraduate students rather than employed individuals. Item number 5 was originally loaded under the “ability to perform primary and secondary task simultaneously”, while in the translated and adapted version, this item was loaded under “general multitasking ability”. The three factors that emerged from the EFA were named similarly to the original measure. *Table 1* also shows that the commonalities of all the items are above than .50, which is an indication of less specific variance among these variables. Furthermore, as Robinson (2018) suggested, the reliability of the shorter scale should be checked carefully to validate the instrument. Proof of validity was carried out by estimating the internal consistency of factor 3 beside the overall measure, while treating the other two factors as a separate dimension in the overall scale.

From *Table 2* we can say that the mean and standard deviations suggest a moderate spread of data. The coefficients of skewness and kurtosis are also in the acceptable range of –1 to +1. The reliability of CSMMI, both overall and on the subscales, was determined by employing Cronbach alpha coefficients. Alpha values are in the acceptable range (Murphy & Davidshofer, 2001; Nunnally, 1978; Salvia, Ysseldyke, & Bolt, 2010). These results have provided evidence for reliability and validity of the translated instrument.

**Table 2:** Descriptive Statistics and Alpha Reliability for all the scores on Scales, Subscales, and Sub Facets of the Study Variables (N=230)

Variables	No of item	Alpha	*M*	*SD*	Potential Range Actual	Skew	Kurtosis
CSMMI^a^	19	.75	55.22	8.99	19–95 30–80	–.04	.39
GMA^b^	7	.73	21.66	4.79	7–35 8–35	–.18	.01
APTMTPTS^c^	10	.72	28.81	5.99	10–50 10–47	–.02	–.16
APPSTS^d^	2	.80	4.74	2.19	2–10 2–10	.56	–.55

*Note. ^a^CSMMI = Communication Specific Multitasking Measurement Instrument; ^b^GMT = General Multitasking Ability; ^c^APTMTPTS= Ability to Perform Two/More Than Two Primary Tasks Simultaneously; ^d^APPSTS = Ability to Perform Primary and Secondary Task Simultaneously*.

## Study II

The main objectives of Study II were to confirm the measurement model through confirmatory factor analysis (CFA) on the factor structure explored in Study I through EFA, both on a larger data of married men and women and according to gender and work status, and also to validate the measure by developing the indices of internal consistency and validity estimates through intra-scale correlation for the translated instrument on the complete data set. The final objective was to investigate gender differences (contrasted group validity) on the translated version of CSMMI by comparing the scores of married men and women.

### Participants and Procedure

To achieve the above-mentioned objectives, data from a larger data set (*N=* 850) of married individuals was collected. The age range of the participants was 23-60 years (*M* = 36.47 & *SD* = 8.83). The sample was comprised of married men and women having children as follows: married working men (*n* = 328), married working women (*n* = 300), and housewives (*n* = 222). The procedural details are like those mentioned under Study I. Data collection procedure also followed that of Study I. Duplication of participation was avoided and it was made sure that all the participants had not taken part in Study I. Informed consent was taken from all participants and they were ensured that confidentiality and anonymity would be upheld.

## Results

### Factorial Validity Through Confirmatory Factor Analysis

A substantial prerequisite for producing high quality data is to create a sound translation and adaptation testing process. This process should acknowledge that measures are sensitive to local background disparities, while remaining equivalent across groups (Swami & Barron, 2019). An EFA-to-CFA approach can be used to estimate the extent to which scores on translated measures are truly invariant across groups. It is important to establish the extent of invariance before comparing latent scores (mean comparisons) across groups. Therefore, to examine the degree to which a measure is invariant across groups (where invariance corresponds to individual items being able to be explained by the same latent factors), CFA should be used as a multi-group invariance method at the configural level (Chen, 2008).

Configural invariance concludes that the number of latent variables and the pattern of loadings of latent variables on indicators are similar across groups, meaning the unconstrained latent model should fit the data well in all groups (Marsh et al., 2009; Swami & Barron, 2019). In the present study, CFA models across different groups of married individuals were estimated to test the variance across groups for the latent construct of multitasking. The aforementioned gender variations in multitasking contributed to the need to assess invariance and to test the functionality of CSMMI items across groups. To achieve the first objective of Study II, CFA was performed to confirm the factor structure explored through EFA in Study I. Factor loading is shown in *Figure 1***.**

*Table 3* shows an estimation of the fit indexes for CSMMI for three models tested on the overall sample and across gender. The results showed model fit indices are above the criteria of .90 the acceptable range given by Kline (2005), as tested against M1 (the overall sample). Using the traditional criteria (Byrne, 2013), chi- square, GFI and RMSEA values indicate good fit of the model to the data (Pearl, 2012). IFI and TLI as goodness of fit indices are also reasonable. This confirms the instrument as a three-dimensional, three-factor construct, as proposed in Study I through EFA on the separate sample of 230 individuals. M2 was assessed to see the variation in the model with respect to gender and the associated indices align with the given criteria of good model fit. The fit indices, chi-square, GFI and RMSEA values are all within the acceptable ranges. This suggests that the translated measure is also valid across the sample of married working men. M3 was estimated and assessed using the data of married women, both working and housewives. Whereas models M4 and M5 examined the invariance across working women and housewives separately. The results showed that these two models fit adequately across the two sample groups of married women. All the fit indices are in the acceptable range, as are the values of chi-square and RMSEA. These results have provided proof of construct validity for the measure of multitasking on the overall data set of married men and women, working and housewives collectively and across separate groups. This measure is also equally valid across gender. In order to estimate reliability and descriptive statistics, the data was analyzed and values of alpha coefficients, mean, standard deviations, skewness, and kurtosis are presented below in *Table 4* below.

**Table 3:** Model Fit Indices for Confirmatory Factor Analysis on CSMMI for the overall sample and across three sample groups (N=850)

	χ ^2a^	*df ^b^*	χ^2^/df	CFI^c^	RMSEA^d^	IFI^e^	TLI^f^	GFI^g^
M1	476.83	146	2.70	.94	.04	.95	.94	.95
M2 (Men *n* = 328)	273.54	148	1.84	.92	.05	.93	.94	.94
M3 (Women n = 522)	415.63	149	1.72	.93	.04	.93	.93	.93
M4 (Housewives n= 222)	215.63	145	2.77	.91	.05	.91	.91	.92
M5 (Working Women n =300)	250.57	149	1.72	.92	.04	.92	.91	.92

*Note. ^a^χ^2^ = chi-square, ^b^df = degree of freedom, ^c^CFI = Comparative Fit Index, ^d^RMSEA = Root Mean Square Error of Approximation, ^e^IFI = Incremental Fit Index, ^f^TLI = Tucker Lewis index, ^g^GFI = Goodness of Fit Index*.

**Table 4:** Descriptive Statistics and Alpha Reliability for all the scores on Scales, Subscales, and Sub Facets of the Study Variables (N=850).

Variables	No of items	Alpha	*M*	*SD*	Potential Range Actual	Skew	Kurtosis
CSMMI^a^	19	.86	55.15	9.49	19–95 19–83	–.29	.27
GMA/ATM^b^	7	.77	20.81	4.99	7–35 8–35	.01	–.29
APTMTPTS^c^	10	.76	29.11	6.38	10–50 10–47	–.23	–.17
APPSTS^d^	2	.73	5.23	2.15	2–10 2–10	.32	–.72

*Note. ^a^CSMMI = Communication Specific Multitasking Measurement Instrument; ^b^GMA/ATM = General Multitasking Ability/Attitudes Towards Multitasking; ^c^APMTPTS= Ability to Perform Two/More than Two Primary Tasks Simultaneously; ^d^APPSTS = Ability to Perform Primary and Secondary Tasks Simultaneously*.

The results of the descriptive statistics in *Table 4* show that the data is normally distributed. Values of alpha coefficients are acceptable as reliability estimates of the translated and adapted version of CSMMI on the large data set of married men and women.

Further construct validity was established through intra-scale correlation i.e., the correlation of total scores between the measure and its three factors and between the factors themselves. *Table 5* shows significant positive correlation between total scores and each factor. A correlation also exists between factors two and three. This pattern of relationship provides evidence for the significane of these factors or subscales within the measure. General Multitasking Ability (GMA) was found to be less significant and did not correlate with the Ability to Perform Primary and Secondary Tasks Simultaneously.

**Table 5:** Construct validity of CSMMI through Intra-scale Correlations between CSMMI and its subscales and between the subscales themselves (N = 850).

Variables	CSMMI^a^	GMA^b^	ATPMTPTS^c^	APPSTS^d^
CSMMI^a^	–			
GMA^b^	.62**	–		
APTMTPTS^c^	.83**	.13**	–	
APPSTS^d^	.52**	.06	.40**	–

Note. ^a^CSMMI = Communication Specific Multitasking Measurement Instrument; ^b^GMA = General Multitasking Ability; ^c^APTMTPTS= Ability to Perform Two/More than Two Primary Tasks Simultaneously; ^d^APPSTS = Ability to Perform Primary and Secondary Tasks Simultaneously. **p < .01.

### Contrasted Group Validity

In order to examine contrasted group validity as a proof of measurement invariance, many researchers (Jørgensen et a., 2018; Picconi et a., 2018) have employed the following approach in validation studies of samples of various groups and across gender. Chen et al. (2019) also assessed the mean differences across two groups of gender (men and women) when looking at levels of education among students to develop a self-efficacy measure. Considering this approach, t-test analysis was carried out to establish the validity of the translated and adapted measure for married men and women separately.

The results of t-test analysis in *Table 6* demonstrates that the differences between the overall scores of men and women on the perceived multitasking ability are insignificant. Whereas significant differences were observed between the factors GMA and APPSTS. The mean values revealed that male participants scored higher on GMA than the female participants, while female participants scored higher on APPSTS than male participants. Overall, mean values were slightly higher among women than among men. Group differences through Analysis of Variance (ANOVA) among the three groups of married individuals (married working men, married working women, and married female housewives) were found to be significant and these results are under review for publication.

**Table 6:** Mean, Standard Deviation, t and d Values for Gender Differences on CSMMI (N =850).

	Married Men (*n* = 328)		Married Women (*n* = 522)				95%CI		
Variables	*M*	*SD*	*M*	*SD*	*T*	*p*	*LL^e^*	*UL ^f^*	Cohen’s *d*
CSMMI^a^	55.29	9.33	55.06	9.60	.34	.73	–1.08	1.54	.02
GMA^b^	21.25	5.25	20.53	4.80	2.05	.04	.03	1.40	.14
APMTPTS^c^	29.05	6.57	29.14	6.26	–.20	.84	–.97	.79	.01
APPSTS^d^	4.98	2.17	5.38	2.13	–2.65	.00	–.69	–.10	.19

*Note. ^a^CSMMI = Communication Specific Multitasking Measurement Instrument; ^b^GMA = General Multitasking Ability; ^c^APTMTPTS= Ability to Perform Two/More Than Two Primary Tasks Simultaneously; ^d^APPSTS = Ability to Perform Primary and Secondary Task Simultaneously; ^e^LL = lower limit; ^f^UL = upper limit*.

## Discussion

In order to achieve the objectives of the present study, two separate studies were conducted. Study I was completed into two phases. In phase I, translation and adaptation of CSMMI into Urdu was completed. Independent bilingual and subject matter experts were involved in the forward and back translation processes. Phase II dealt with the empirical validation (through EFA) of the translated and adapted instrument. The data was checked for appropriateness by the Kaiser-Meyer-Olkin Measure of Sampling Adequacy and Bartlett’s Test of Sphericity. The results provided evidence for the adequacy of the data as per the criteria (Tabachnick & Fidell, 2013). The results of Bartlett’s Test of Sphericity advocated for an EFA procedure as per Field (2009) criteria. Varimax rotation was used (Kahan, 2006), as it explained the maximum amount of variance (Tabachnik & Fidell, 2007). This method gave 3 factors with eigenvalues greater than 1. These three factors were considered as the three dimensions and subscales of the translated measure, explaining 40% of the variance.

CFA was applied after EFA as the stand-alone CFA model showed poor and unidentified model fitness for CSMMI on the data of married working individuals. Swami and Barron’s (2019) approach of applying a EFA-to-CFA method to estimate the extent to which scores on translated measures are truly invariant across groups was then followed. This was done by comparison of latent scores (mean comparisons) for the construct of multitasking across groups. The results of EFA were confirmed through CFA. The factor structure explored in Study I was confirmed by the results derived from the larger data set in Study II. The results of Study II showed three factor model as reliable, valid, and applicable for married individuals including men and women (both working & housewives). Hence, this has fulfilled the need for a sound and stable measure of multitasking.

The results provide strong proof of empirical and construct validity for a Pakistani version of CSMMI. The original author, Kushniryk (2008), of the instrument also suggested to establish the validity of the measure across different cultures. The findings of this study have made this a reality and have thereby extended the validity of the measure across different populations and cultural contexts.

Researchers often equate differences in groups with psychological variances. For an effective cross-cultural comparison, it is necessary to translate measures and adapt them appropriately so that they can be administered to another culture or group of people. When using this strategy, researchers often assume that the instrument examines the same psychological construct in all groups. They run CFA models with sample data collected from a population to test that the items of a scale are good indicators of a given latent construct (Milfont & Fischer, 2010) for the overall sample data or across groups. In comparing groups, an assumption is made that the measure studies the same psychological construct in all groups. When testing for invariance in cross-cultural research, member of different groups (e.g. men and women) attribute similar meanings to the given instrument (Fischer et al., 2009; Gouveia, et al., 2009; Milfont et al., 2006; Milfont & Fischer, 2010). In the present study, testing of the model across gender and working status of married individuals showed that all the model fit indices were in the acceptable ranges. This suggested that the translated and adapted version of CSMMI is equally valid for married working men, married working women and female housewives. From these results, it is concluded that the number of latent variables and the pattern of loadings of these latent variables on indicators is similar across groups, meaning that the unconstrained latent models provide a good fit for the data in all groups (Marsh et al., 2009; Swami & Barron, 2019), before comparing these groups through further statistical techniques such as mean differences across these groups.

To establish the contrasted group validity, group differences across gender were investigated. Although the results indicated insignificant gender differences overall, differences were observed between subscales i.e., General Multitasking Ability (GMA), on which men scored higher than women, and the Ability to Perform Primary and Secondary Tasks Simultaneously (APPSTS), in which women scored higher than men. The findings are consistent with past literature (Bianchi, John, & Milkie, 2006; Bianchi & Wight 2010; Floro & Miles, 2003; Galinsky at al. 2005; Kushniryk, 2008; Mantyla, 2013; Offer & Schneider 2011). While regarding the dynamic construct of multitasking, Mantyla (2013) suggested that gender differences with regards to multitasking should be interpreted carefully and thoughtfully. The empirical evidence for disparities between genders suggests invariance in multitasking in terms of gender differences (Strayer et al., 2013), wherein differences in executive attention most likely influence multitasking abilities (Strayer & Watson, 2012). A possible reason for the insignificant gender differences may be the inequivalent sample sizes of men and women. However, it might be important here to share that the results of ANOVA conducted on the three sample groups of married individuals showed significant mean differences. These results are reported in another manuscript under evaluation for publication.

The results of Study I in Table 2 provided evidence for the normality of the data and the reliability of the translated and adapted measure of multitasking for Pakistan as a representative of collectivist Asian culture. These results are consistent with the previous studies using this instrument (Kalsoom & Kamal, 2018; Kalsoom & Kamal, 2020), which have also reported high levels of reliability and validity for this measure. In this study, proof of validity and reliability is also reported in relation to gender roles, multitasking preferences and marital adjustment, along with perceived multitasking ability (by using the translated and adapted version of CSMMI). This level of reliability and validity was also reported for the translated and adopted version of CSMMI into Chinese language (Widyahastuti & Anwar, 2017). This study further deepened the validity of this measure by considering reliability coefficients and intra-scale correlations. These results sufficiently provided evidence for internal consistency and association of the construct with its subscales on the sample of married men and women (working and housewives). This is also consistent with previous studies (Kalsoom & Kamal, 2018; Kalsoom & Kamal, 2020). The instrument has also been validated using the Big Five Personality Dimensions i.e., extraversion, conscientiousness, openness and neuroticism (Widyahastuti & Anwar, 2017). Such validations of CSMMI are important for the generalizability and applicability of this measure across populations and other cultural contexts. More specifically, they are important with respect to the collectivist Asian culture of Pakistan and others like India, Bangladesh, Afghanistan, and Iran, where the language and context is adequately applicable.

## Conclusion

These findings are important in extending the validity of the CSMMI that was originally developed in English and herein translated and adapted into Urdu. The newly established factor structures provide a modified model of CSMMI for future research, not only in Pakistan but in other similar countries where the English language is a barrier (e.g. India and Bangladesh). The results of this study have provided empirical evidence for all the items in the original measure, as well as providing empirical support for bridging the gap in the literature between the psychology of multitasking, gender differences and collectivist cultures. Theoretically, the findings of this study have provided further evidence for perceived multitasking ability as a multifactorial construct in relation to communication. This provides pragmatic grounds for the utility and adequacy of the measure across different language and cultural contexts. Therefore, the adapted measure is a useful addition to the evolving study of the construct of multitasking, and has provided good reason to believe that the use of this measure in future research studies across different groups and cultures will be equally as reliable and valid.

## Limitations and Suggestions

This study is an adding to the understanding and validation of the construct of multitasking in relation to the working population of Pakistan as a representative of collectivist Asian cultures. The data was collected from married men and women with children from the two biggest cities in Pakistan. This may be a generalizing limitation. However, data was collected from a real-life work setting, and this may be a strength for validating the measure of multitasking, whereas in the original study, a sample of university students was selected to develop and validate the measure.

In this study, multitasking was considered a self-perceived ability by employing the self-reported data of married men and women. However, future studies should use this measure alongside other data collection methods, such as in-depth interviews, as this may yield more distinct and discrete features of multitasking that can be incorporated into the measure.

Overall, the results of the study provided the strong evidence for empirical and construct validity and reliability of the measure. This could be extended by establishing evidence for convergent and discriminant validity.
